# Optimization of Supercritical CO_2_ Extraction of Fish Oil from Viscera of African Catfish (*Clarias gariepinus*)

**DOI:** 10.3390/ijms130911312

**Published:** 2012-09-11

**Authors:** Mohamed Zaidul Islam Sarker, Jinap Selamat, Abu Sayem Md. Ahsan Habib, Sahena Ferdosh, Mohamed Jahurul Haque Akanda, Juliana Mohamed Jaffri

**Affiliations:** 1 Department of Pharmaceutical Technology, Faculty of Pharmacy, International Islamic University, Kuantan Campus, Kuantan 25200, Pahang, Malaysia; E-Mail: juliana@iium.edu.my; 2 Centre of Excellence for Food Safety Research (CEFSR), Universiti Putra Malaysia, Serdang 43400, Selangor, Malaysia; E-Mail: issuebau@gmail.com; 3 Institute of Tropical Agriculture, Universiti Putra Malaysia, 43400 Serdang, Selangor, Malaysia; 4 School of Industrial Technology, Universiti Sains Malaysia, Minden, Penang 11800, Malaysia; E-Mails: sfshila@gmail.com (S.F.); jahurulhaque@yahoo.com (M.J.H.A.)

**Keywords:** supercritical fluid extraction, catfish fish oil, viscera, response surface methodology

## Abstract

Fish oil was extracted from the viscera of African Catfish using supercritical carbon dioxide (SC-CO_2_). A Central Composite Design of Response Surface methodology (RSM) was employed to optimize the SC-CO_2_ extraction parameters. The oil yield (*Y*) as response variable was executed against the four independent variables, namely pressure, temperature, flow rate and soaking time. The oil yield varied with the linear, quadratic and interaction of pressure, temperature, flow rate and soaking time. Optimum points were observed within the variables of temperature from 35 °C to 80 °C, pressure from 10 MPa to 40 MPa, flow rate from 1 mL/min to 3 mL/min and soaking time from 1 h to 4 h. However, the extraction parameters were found to be optimized at temperature 57.5 °C, pressure 40 MPa, flow rate 2.0 mL/min and soaking time 2.5 h. At this optimized condition, the highest oil yields were found to be 67.0% (g oil/100 g sample on dry basis) in the viscera of catfish which was reasonable to the yields of 78.0% extracted using the Soxhlet method.

## 1. Introduction

African catfish (*Clarias gariepinus*) occupy a large area in aquaculture in Africa [1]. Recently it has spread in Europe and southern Asia for its great economic interest: faster growth rate, omnivorous feeding habit, and high resistance to environmental stress [1–3]. It is also considered one of the most important tropical catfish species for aquaculture [4]. In Malaysia, it is called ikan keli and is the most preferred fresh water fish among Malaysians. Fish oils are rich sources of natural bioactive lipid components. These lipid components are commercially used in the pharmaceutical and food industries and as human health supplements. Moreover, fish is being considered an important diet due to its polyunsaturated fatty acids (PUFAs) content. Its curative and preventive effects are well recognized in treating cardiovascular diseases, autoimmune disorders, various kinds of inflammation [5], cancers and their effect in the neurodevelopment of infants [6]. Therefore, the consumption of fish and fish products is increasing day by day all over the world. Moreover, several food process industries supply their products under different brands throughout the year for those who are not habituated to the direct consumption of fish.

Hence, the by-product generation, including skin, viscera, head, scales and bones from the fish process industries has increased, mostly considered previously as worthless garbage and discarded without any attempt at recovery [7,8]. It is estimated that annually 20 million tonnes or equivalent to 25% of the total production of fish is discarded as by-products or waste materials [9]. As a result, these huge amounts of by-products create both disposal as well as pollution problems [10]. However, these materials can be a potential source of enzymes and fats [8,11,12], protease producing bacteria [13], lactic acid fermentation media [14] as well as protein and bioactive lipid components. Depending on the species, food habit, geographical location, catch season and maturity, the oil content of fish waste lies between 1.4% and 40.1% [15].

Many researchers have reported the extraction, fractionation and purification of fish oils using various conventional methods, such as hydraulic pressing, vacuum distillation, urea crystallization, hexane extraction, and conventional crystallization. The major disadvantages of these methods are the requirement of high temperatures that affect the nutritional quality of the fish oils, degradation of the heat sensitive labile natural compounds, and toxic solvent left in the final products, all of which have adverse human health effects [16,17]. Moreover, a large number of studies have been carried out on one salt water fish species for lipid extraction using various methods, while little attention has been paid to the extraction of lipid from fresh water fish species. Supercritical fluid extraction (SFE) is the method of choice for the extraction and fractionation of edible natural oils from various sources. To date, numerous studies have been carried out for the extraction of fish oil fatty acids using the SFE technique [18–23]. Over the last 20 years, SFE has been acknowledged as a promising alternative to the organic solvent extraction method in the field of natural fats and oils. The major merits of the SFE method is the lack of solvent residue left in the final products and better retention of valuable components [24–29]. Carbon dioxide is used as a solvent due to its nontoxic, non-flammable, inexpensive, and cleanness, which offer great opportunities for complex separation processes.

The waste materials from the African Catfish, mainly the viscera, can be a reliable source of raw material throughout the year for the extraction of lipid at an industrial scale as it has minimal/negligible seasonal variation regarding chemical composition [4]. Therefore, the objective of this study was to optimize the SC-CO_2_ extraction of fish oil from the wastes such as viscera.

## 2. Results and Discussion

### 2.1. Effect of SFE Parameters on the Oil Yield

The full experimental design and corresponding data obtained are shown in Table 1, where the highest yield was obtained at run order 10, followed by 29, 19, 13 and 30 (Table 1). The combined effect of temperature, pressure, flow rate and soaking time compensated gaining the density of supercritical CO_2_, which meant increased solubility of the solute in the solvent. Temelli *et al*. [30,31] reported that the impact of temperature is dependent on competing parameters; the CO_2_ density decreases with temperature while the vapor pressure of the solutes increases, leading to the well-known crossover phenomenon for solubility isotherms. Thus, the resultant impact of temperature on solubility is dictated by whichever parameter is greater at a given pressure. In addition, diffusivity increases with temperature enhancing mass transfer kinetics during extraction [30–32]. The statements are in agreement with our observation of the effect of pressure, temperature and flow rate on the recovery of the total oil yield, as shown in Table 1. In general, extraction yield increases with pressure, due to an increase in lipid solubility in supercritical CO_2_, and based on an increase in CO_2_ density. Thus, the highest yield (67.0%) was obtained for the combined effect of 40 MPa, 57.5 °C, 2 mL/min and a soaking time at 2.5 h. This yield was reasonable to the yield (78.0 g/100 g sample, on dry basis) extracted using Soxhlet method.

### 2.2. Fitting the Response Surface Models

In order to determine accuracy, the coefficient of determination (*R*^2^) was calculated for each run. For a good fit model, *R*^2^ should be at least 0.80 [33], and in our significant model it was found to be 0.996. The regression coefficient values of the corresponding variables for this experiment are presented in equation 1. The oil yield (*Y*) was estimated by the second order polynomial equation shown below:

(1) $$\begin{array}{l} Y=47.313+(-0.122)X_{1}+(-0.131)X_{2}+4.739X_{3}+(-1.759)X_{4}+(-0.001){X_{1}}^{2}+0.004{X_{2}}^{2}+\\ (-1.300){X_{3}}^{2}+0.400{X_{4}}^{2}+0.053X_{2}X_{3}+0.007X_{1}X_{2}+0.055X_{1}X_{3}+0.003X_{1}X_{4}+0.020X_{2}X_{4}+\\ (-0.167)X_{3}X_{4} \end{array}$$

Where, *Y* represents the oil yield of applied temperature (*X*_1_), pressure (*X*_2_), flow rate (*X*_3_) and soaking time (*X*_4_). From Equation 1, it was observed that the linear terms: temperature, pressure and soaking time had negative effects whereas flow rate had a positive effect on oil yield. However, all of the independent variables e.g., temperature, pressure flow rate and soaking time, were the most significant parameters in SC-CO_2_ extraction. Two quadratic terms (*X*_2_^2^ and *X*_4_^2^) had positive and two (*X*_1_^2^ and *X*_3_^2^) had negative effects on the yield. On the other hand, the interaction terms, *X*_2_*X_3_, *X*_1_*X_2_, *X*_1_*X*_3_, *X*_1_*X*_4_ and *X*_2_*X_4_ had positive effects whereas *X*_3_*X*_4_ had a negative effect on the yield. However, the optimum conditions were obtained at 57.5 °C, 40.0 MPa, 2 mL/min and 2.5 h for temperature, pressure, flow rate and soaking time, respectively, with the maximum oil yield of 67.0%.

### 2.3. Analysis of Response Surface

**Figure 1:**
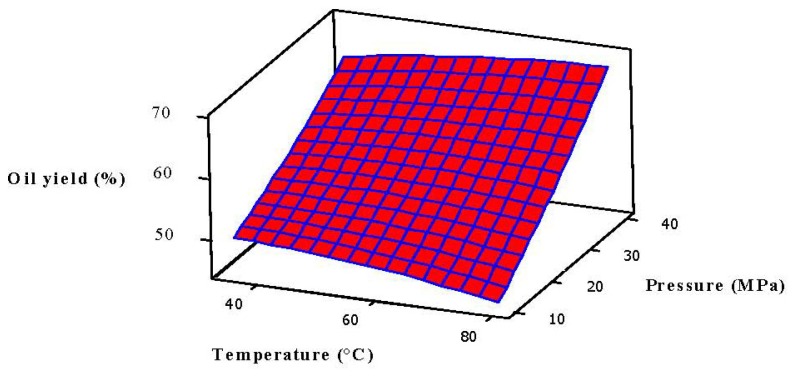
Response surface plot for the oil yield (*Y*) as a function of temperature and pressure at a fixed CO_2_ flow rate of 2 mL/min and soaking time of 2.5 h.

For a better understanding and representing of the significant (*p* < 0.05) statistical interaction of factors in response, a three dimensional (3D) surface data plot is highly recommended by Xu *et al.* [34], which is shown in Figure 1. It was sketched by keeping the temperature and pressure constants low, following which the oil yield was also found to be low. The yield significantly increased at relatively high pressure (>28 MPa) and medium to high temperature from 55 °C to 65 °C. The increasing effect of temperature and pressure was statistically significant (*p* < 0.05) on oil yield up to 65 °C. Above 65 °C, the oil yield gradually started to decrease with pressure, which is probably due to the reduction of CO_2_ density and solvation power of the SC-CO_2_. However, the effect of pressure changes (10 MPa to 40 MPa) on oil yield had a more noticeable and significant effect than that of temperature. Pressure elevation at a given temperature enhances the solubility of the oil in SC-CO_2_, which might improve the total oil yield [35]. Similar trends were also observed in the extraction of silkworm pupal oil using SC-CO_2_ [36,37]. Authors concluded that temperature has a negative effect on yield after a certain limit.

**Figure 2:**
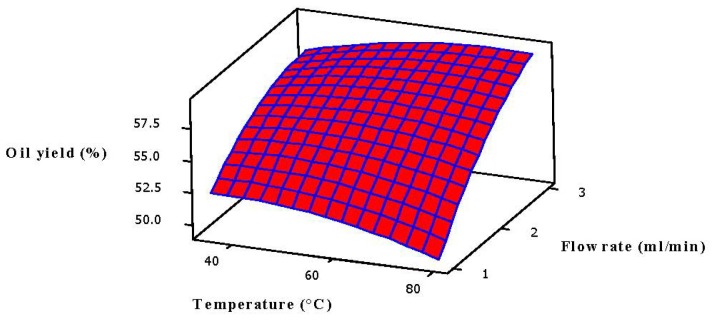
Response surface plot for the oil yield (*Y*) as a function of temperature and flow rate at a fixed pressure 25 MPa and a soaking time of 2.5 h.

According to Figures 1 and 2, pressure and flow rate had similar effects on oil yield at constant temperature, thus the temperature interacted with pressure and flow rate. The solvent density, vapor pressure, and the solubility mode of the solute depend on these two variables. Consequently, it is very difficult to predict and elucidate the effect of temperature on oil yield. At constant pressure, the oil yield increased significantly with both temperature and flow rate (Figure 2), since high temperature decreases the density of CO_2_ and thus leads to an increase in vapor pressure of the solutes that improves the solubility as well as the mass transfer rate of solute, resulting in easier extraction of the desired compounds [25,36,37].

**Figure 3:**
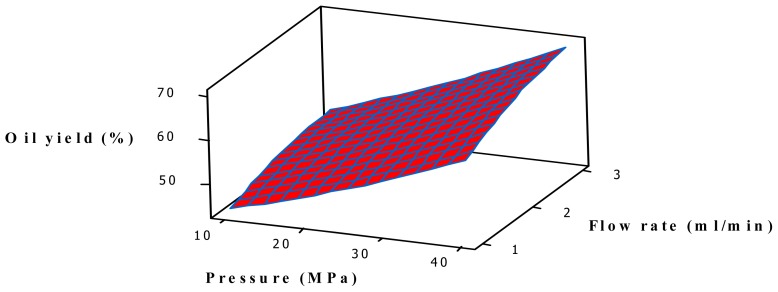
Response surface plot for the oil yield (*Y*) as a function of pressure and flow rate at a fixed temperature of 57.50 °C and soaking time of 2.5 h.

Figure 3 shows a significant interaction between pressure and flow rate on oil yield. Raising the pressure at constant temperature increased the density of CO_2_ as well as the extraction efficiency of the solutes. However, performing the extraction using SC-CO_2_ at high pressure is not always advisable, as at high pressure levels where the highly compressed CO_2_ may provoke a complexity in the extraction by introducing a repulse reaction between the solute and solvent [38]. Therefore, the combined effect of pressure and flow rate was not as significant as shown in Figure 3, while the flow rate was interacting with the temperature. Moreover, this leads to a decrease in yield at the time of fluid decompression, either by increasing analyte loss or by using an elevated pressure drop through the extraction cell [38].

The maximum oil yield (67.0%) was obtained from viscera at optimum conditions. Our results are similar to the results of Rubio-Rodríguez *et al.* [39], who found 63% hake oil from offcuts using supercritical fluid extraction. In another study, about 53.2% of oil was reported from the skin of Indian mackerel in using various techniques of supercritical fluid extraction [23].

## 3. Experimental Section

### 3.1. Materials

Fresh African Catfish (*Clarias gariepinus*) samples were collected from a local market in Malaysia. A cylinder of carbon dioxide with a purity of 99.99% was purchased from Malaysian Oxygen Ltd. Kuala Lumpur, Malaysia and all other solvents and chemicals used in this experiment were analytical grade and obtained in Malaysia.

### 3.2. Sample Preparation for Experiments

The samples were immediately de-headed and washed with copious amounts of fresh water to separate the viscera. The viscera were then stored overnight in a freezer at −18 °C, and then freeze dried (Model: LABCONCO, USA) at a constant drying temperature of −47 °C and vacuumed at 0.133 bar. The dried samples were ground using a blender and stored in an airtight glass bottle in a coldroom at 6 °C pending laboratory use.

### 3.3. Moisture Content Determination

The moisture content of the dried sample was determined by the oven dry method [40]. Five grams of finely ground sample was place in pre-weighted ceramic crucibles before being put into the oven. The temperature of the oven was set at 105 °C, and the heating process continued until constant weights of the samples were achieved. Then, the crucibles were transferred to a desecrator to cool before reweighing. The difference of the two weights (initial and final) indicated the moisture content and it was found to be 3.95% in viscera.

### 3.4. Soxhlet Extraction

Total lipid content of the catfish viscera was determined by Soxhlet extraction method. Five grams of finely ground dried samples were extracted with 200 mL of petroleum ether with three replications over an extraction period of 8 h. Extra water and petroleum ether residue in the extracted oils were evaporated using a rotary evaporator (Heidolph, Germany) at a temperature of 45 °C. The evaporated sample was then dried in an oven at 45 °C for 1 h. Total lipid content of the catfish viscera was 78 ± 0.6% based on the dry weight of the sample.

### 3.5. Apparatus and Procedure of Supercritical Fluid Extraction

All the runs were carried out in a supercritical fluid apparatus (Model PU-1580, Jasco Corporation, Tokyo). For each trial, 5 g of dry sample was loaded into a 10 mL extraction vessel (model Ev-3, Jasco Corporation, Tokyo), and then placed into an external water bath at a temperature ranging from 35 °C to 80 °C. Then, the valve of the CO_2_ cylinder was opened and the CO_2_ allowed to circulate through the cooling jacket of the chiller to cool before reaching the extraction vessel at a constant flow rate ranging from 1 mL/min to 3 mL/min (Model 631 D, Tech-Lab Manufacturing sdn. Bhd., Selangor, Malaysia), where CO_2_ gas was converted to liquid form. After reaching the desired pressure in the extraction vessel, the CO_2_ valve was closed for a certain period of time to soak the sample in pure CO_2_: regarded as the “soaking time” for this experiment. A back pressure regulator (BPR) (model BP-1580-81, V, Jasco Corporation, Tokyo) was used to control the system pressure and separate the CO_2_ from the extract. Then, the CO_2_ valve was opened again during continuous extraction at constant pressure, temperature and flow rate. At each condition, experiments were conducted in duplicate, and each yield was the mean of duplicate measurements. Finally, the yield trap was collected and stored at −18 °C for further analysis.

### 3.6. Experimental Design

A central composite design consisting of 30 experimental runs with six replications at the central points were employed to optimize the extraction variables, namely temperature, pressure, flow rate and soaking time. The creation of design matrix, experimental data analysis and optimization were all undertaken using Minitab v.14 statistical software. The polynomial equation represents all possible combinations of the extracting variables (*X*_1_, *X*_2_, *X*_3_ and *X*_4_) of their main, quadratic as well as the interacting effects on the response variable of oil yield (*Y*). A preliminary study was conducted to select the range values of the parameters (Table 2). More emphasis was given for the selection of temperature level: the lower limit was 35 °C, just above the critical points of CO_2_ (31.1 °C) and the upper limit was not more than 80 °C to save the thermally sensitive compounds from thermal degradation [41]. All the design points were performed three times except the centre point. Experiments were run in randomized order to minimize the effect of unexplained variability induced by extraneous factors. The polynomial regression equation presented below was used for predicting the response variable (*Y*).

(2) $$\text{Y}=\beta_{0}+\sum_{i=1}^{4}\beta_{i}{\text{X}}_{i}+\sum_{i=1}^{4}\beta_{ii}{{\text{X}}_{i}}^{2}+\sum \sum_{i\langle j=1}^{4}\beta_{ij}{\text{X}}_{i}{\text{X}}_{j}$$

Where, *Y* is the response (percentage of oil yield) *β*_0_ is a constant and *β_i_*, *β_ii_*, *β_ij_* are the linear, quadratic and interaction terms, respectively. X*_i_* and X*_j_* are the levels of independent variables.

## 4. Conclusions

At the optimized condition, the SFE extracted oil yield was 67.0% from the viscera on a dry weight basis, which was reasonable when compared with the yield extracted using the Soxhlet method. However, at the optimized conditions, all the individual variables e.g., pressure, temperature, flow rate and soaking time were the most significant linear terms and the system was very sensitive to minimal changes in those variables. By contrast, the quadratic terms: the flow rate and soaking time were the most significant whether positive or negative. On the other hand, in the interactions no (0) effect was found between pressure and flow rate; however, all the interaction terms had a positive effect except in the interaction between flow rate and soaking time.

## Figures and Tables

**Table 1:** Central Composite Design matrix of factors and the responses of oil yield for African Catfish viscera.

Run Order	Blocks	Temperature (°C)	Pressure (MPa)	Flow rate (mL/min)	Soaking time (h)	Oil yield (%)	Predicted yield (%)
1	3	57.50	25.0	2.0	4.00	58.2 ± 0.34	58.33
2	3	35.00	25.0	2.0	2.50	55.9 ± 0.43	56.00
3	3	57.50	25.0	3.0	2.50	59.0 ± 0.40	58.92
4	3	57.50	25.0	2.0	2.50	56.5 ± 0.22	56.57
5	3	57.50	25.0	1.0	2.50	51.0 ± 0.38	51.62
6	3	57.50	10.0	2.0	2.50	47.5 ± 0.39	48.22
7	3	80.00	25.0	2.0	2.50	55.2 ± 0.25	55.63
8	3	57.50	25.0	2.0	1.00	56.2 ± 0.39	56.60
9	3	57.50	25.0	2.0	2.50	56.6 ± 0.37	56.57
10	3	57.50	40.0	2.0	2.50	67.0 ± 0.17	66.82
11	1	68.75	32.5	1.5	3.25	59.8 ± 0.21	59.93
12	1	68.75	17.5	1.5	1.75	49.0 ± 0.42	48.87
13	1	46.25	32.5	2.5	3.25	62.9 ± 0.26	62.87
14	1	68.75	17.5	2.5	3.25	53.5 ± 0.41	53.43
15	1	57.50	25.0	2.0	2.50	56.5 ± 0.47	56.57
16	1	57.50	25.0	2.0	2.50	56.6 ± 0.37	56.57
17	1	46.25	17.5	2.5	1.75	53.9 ± 0.35	53.60
18	1	46.25	17.5	1.5	3.25	51.6 ± 0.27	51.57
19	1	68.75	32.5	2.5	1.75	63.6 ± 0.20	63.47
20	1	46.25	32.5	1.5	1.75	58.5 ± 0.32	58.40
21	2	46.25	32.5	1.5	3.25	59.7 ± 0.22	59.57
22	2	46.25	17.5	1.5	1.75	51.3 ± 0.29	50.85
23	2	68.75	17.5	1.5	3.25	50.4 ± 0.44	49.68
24	2	57.50	25.0	2.0	2.50	56.6 ± 0.37	56.57
25	2	57.50	25.0	2.0	2.50	56.6 ± 0.37	56.57
26	2	68.75	32.5	1.5	1.75	59.0 ± 0.45	58.67
27	2	46.25	17.5	2.5	3.25	54.1 ± 0.56	54.07
28	2	68.75	17.5	2.5	1.75	53.1 ± 0.59	52.87
29	2	68.75	32.5	2.5	3.25	64.4 ± 0.19	64.48
30	2	46.25	32.5	2.5	1.75	61.6 ± 0.26	61.95

**Table 2:** Experimental ranges of the independent variables used in the Central Composite Design (CCD) for the oil yield.

Factors	Codes	Levels		

		−1	0	+1
Temperature (°C)	*X*_1_	35	57.5	80
Pressure (MPa)	*X*_2_	10	25	40
Flow rate (mL/min)	*X*_3_	1	2	3
Soaking time (hr.)	*X*_4_	1	2.5	4
